# Modulation of Intestinal Epithelial Permeability via Protease-Activated Receptor-2-Induced Autophagy

**DOI:** 10.3390/cells11050878

**Published:** 2022-03-03

**Authors:** Yuju Kim, Yunna Lee, Gwangbeom Heo, Sihyun Jeong, Soyeong Park, Jin-Wook Yoo, Yunjin Jung, Eunok Im

**Affiliations:** 1 Department of Pharmacy, College of Pharmacy, Pusan National University, Busan 46241, Korea; kyuju96@pusan.ac.kr (Y.K.); yunnalee@pusan.ac.kr (Y.L.); gbheo@pusan.ac.kr (G.H.); jeongsh@pusan.ac.kr (S.J.); sypark@pusan.ac.kr (S.P.); 2 Department of Manufacturing Pharmacy, College of Pharmacy, Pusan National University, Busan 46241, Korea; jinwook@pusan.ac.kr (J.-W.Y.); jungy@pusan.ac.kr (Y.J.)

**Keywords:** PAR2, autophagy, tight junction, permeability, β-arrestin

## Abstract

Protease-activated receptor 2 (PAR2) alleviates intestinal inflammation by upregulating autophagy. PAR2 also modulates tight junctions through β-arrestin signaling. Therefore, we investigated the effect of PAR2-induced autophagy on intestinal epithelial tight junctions and permeability. RT-PCR, Western blot analysis, and immunoprecipitation were performed to investigate the underlying molecular mechanisms by which PAR2 regulates autophagy and intestinal epithelial tight junctions. Inhibition of PAR2 by GB83, a PAR2 antagonist, decreased the expression of autophagy-related and tight-junction-related factors in Caco-2 cells. Moreover, inhibition of PAR2 decreased intestinal transepithelial electrical resistance. When PAR2 was activated, intestinal permeability was maintained, but when autophagy was suppressed by chloroquine, intestinal permeability was significantly increased. In addition, the prolongation of ERK1/2 phosphorylation by PAR2–ERK1/2–β-arrestin assembly was reduced under autophagy inhibition conditions. Therefore, PAR2 induces autophagy to regulate intestinal epithelial permeability, suggesting that it is related to the β-arrestin–ERK1/2 pathway. In conclusion, regulating intestinal epithelial permeability through PAR2-induced autophagy can help maintain mucosal barrier integrity. Therefore, these findings suggest that the regulation of PAR2 can be a suitable strategy to treat intestinal diseases caused by permeability dysfunction.

## 1. Introduction

Autophagy is the process of degradation of cellular components at the lysosome and can be classified as macroautophagy, microautophagy, or chaperone-mediated autophagy. In general, the term “autophagy” refers to macroautophagy (hereafter referred to as autophagy) [1,2]. Autophagy involves engulfing and degrading cytoplasmic components through a double-membrane organelle called the autophagosome [3]. Autophagy plays an important role in maintaining intestinal homeostasis through intestinal immune response and microbial antigen regulation [4]. Autophagy dysfunction leads to several inflammatory diseases, including inflammatory bowel disease [5]. In addition, pharmacological activation of mucosal autophagy alleviates intestinal inflammation [6]. Autophagy is closely associated with the intestinal epithelium, suggesting that autophagy plays an important role in intestinal mucosal integrity.

The intestinal epithelium, which constitutes the barrier, not only absorbs nutrients into the body, but also prevents the inflow of external harmful substances such as antigens and pathogens. Tight junctions (TJs) between intestinal epithelial cells act as a physical barrier by regulating the paracellular movement of molecules [7]. TJs are variable structures that are reassembled by various external stimuli and are involved in intercellular adhesion and intracellular signaling [8]. TJs are formed in epithelial cells by proteins such as claudins, occludins (which are functionally important), zonula occludens (ZOs), cingulin (which are intracellular plaque proteins), tricellulin, and junctional adhesion molecule-A [9].

Intestinal permeability is defined as the functional characteristic of the intestinal barrier that is regulated by a physical barrier composed of TJs, adherens junctions, and desmosomes formed in a monolayer of intestinal epithelial cells [10,11]. TJs are important for controlling permeability as they form a seal between the epithelial cells. Claudin-1 is essential for general barrier function, whereas claudin-2 is permeable to both water and cations through pore formation and is overexpressed in leaky epithelial tissues [12]. The deficiency of occludins does not affect intestinal permeability function [13]. ZO-1 is a cytoskeletal linker protein that interacts with cytoplasmic proteins by forming strong cross-links. Disruption of the TJs destroys the integrity of the intestinal mucosa [14].

Intestinal permeability dysfunction causes several diseases as it allows external environmental factors to enter the intestine [15]. Tumor necrosis factor (TNF) induces the phosphorylation of intestinal epithelial myosin light chain, thereby increasing intestinal permeability, which induces diarrhea [16]. Intestinal pathogens and food allergies also modulate TJs and increase intestinal permeability. Moreover, intestinal permeability increases in patients with Crohn’s disease (CD). Increased intestinal permeability can help predict CD recurrence in patients with inactive diseases [17].

Protease-activated receptor 2 (PAR2) is a member of the PARs, belonging to the G-protein-coupled receptor family [18]. The PARs are seven transmembrane domain receptors that are activated by specific proteolytic cleavage of their extracellular N-terminus by proteases [19]. PAR2 is canonically activated by trypsin cleavage of SKGR34 S35LIGKV and noncanonically activated by other serine proteases or coagulation factors such as VIIa and Xa [20]. PAR2 is expressed in a variety of cells in the gastrointestinal (GI) tract, including epithelial cells, smooth muscle cells, fibroblasts, and some immune cells [19]. Furthermore, PAR2 is activated on both apical and basolateral sides of the intestine, but the receptors on each side induce distinct PAR2 signaling pathways [21]. These findings suggest that PAR2 is constantly activated in the gut and plays multiple roles.

Activated PAR2 has two signaling pathways. Canonical signaling of PAR2 induces phospholipase C/Ca^2+^/protein kinase C signaling by binding to the G-protein and activation of nuclear extracellular signal-regulated kinase (ERK1/2) via Ras [22]. PAR2 also has a signaling pathway through β-arrestin. β-arrestin negatively regulates G-protein signaling, mediates PAR2 internalization, and then assembles the PAR2–β-arrestin–Raf-1–mitogen-activated protein kinase (MEK-1)–ERK1/2 complex. β-arrestin signaling prolongs ERK1/2 activity in the cytoplasm by delaying the translocation of ERK1/2 into the nucleus [23].

A previous study reported that PAR2 deficiency exacerbated colitis by suppressing autophagy [24]. Other studies have shown that activation of PAR2 decreases the expression of TJ proteins and therefore increases mucosal permeability in the intestine and kidney cells [25,26]. Starvation-induced autophagy increases the expression levels of TJ proteins in intestinal epithelial cells [27], suggesting a close relationship between autophagy and mucosal membrane integrity. Therefore, we hypothesized that the activation of PAR2 enhances autophagy in the intestinal mucosa and that increased autophagy signals elevate the expression levels of TJ proteins to downregulate intestinal inflammation through ERK1/2- and β-arrestin-dependent mechanisms. The goals of this study were: (1) to determine whether activation of PAR2 induces autophagy; (2) to investigate the effect of autophagy on TJ protein expression; (3) to examine the prolonged activity of ERK1/2 and its assembly with β-arrestin via PAR2 activation; and (4) to determine whether autophagic cell death is a prerequisite for PAR2-mediated mucosal barrier integrity.

## 2. Materials and Methods

### 2.1. Cell Culture and Treatment

Human colorectal adenocarcinoma cells (Caco-2) were purchased from the American Type Culture Collection (Manassas, VA, USA). Cells were cultured in Dulbecco’s modified Eagle’s medium (DMEM) with high glucose (4.5 g/L; Hyclone Laboratories, Logan, UT, USA), 10% heat-inactivated fetal bovine serum (FBS, Hyclone Laboratories), and 100 U/mL penicillin–streptomycin solution (Hyclone Laboratories) at 37 °C in a 5% CO_2_ atmosphere.

The Caco-2 cells were pretreated with serum-free medium (SFM) for 24 h for equalization. GB83 (Axon Medchem, Groningen, The Netherlands), a PAR2 antagonist, was dissolved in dimethyl sulfoxide (DMSO; Duchefa, Haarlem, The Netherlands) and treated at a concentration of 10 μM in Caco-2 cells. PAR2-activating peptide (PAR2-AP; Peptron, Daejeon, Korea) was dissolved in distilled water and cells were treated at a concentration of 100 μM. The cytokine cocktail (CC) was a mixture of human interleukin (IL)-1β, TNF-α, and interferon (IFN)-γ (PeproTech Inc., Rocky, Hill, NJ, USA), with working concentrations of 1, 20, and 10 ng/mL, respectively. Chloroquine (CQ, Sigma-Aldrich, St. Louis, MO, USA) was dissolved in distilled water to a final concentration of 100 μM. Cells were treated with GB83 for 24 h, with CC for 6 h, with PAR2 for 6 or 24 h, and with CQ for 24 h.

### 2.2. Transfection

Cells were transfected at 80–90% confluence using Lipofectamine 3000 or RNAiMax (Invitrogen Life Technologies, Carlsbad, CA, USA). Transfection was performed in accordance with the manufacturer’s guidelines. To knock down (KD) PAR2, transfection was performed using PAR2 siRNA (Bioneer, Daejeon, Korea) and a negative control (NC) was prepared using siRNA (Bioneer). Cells were transfected with 30 pmol PAR2 siRNA or NC siRNA and incubated for 48 h. For PAR2 overexpression (OE), cells were transfected with 5 µg of pcDNA3-hPar2 plasmid (Addgene, plasmid #53228, Watertown, MA, USA) or control pcDNA3.1 plasmid (Promega, Fitchburg, WI, USA) and incubated for 48 h.

### 2.3. Reverse-Transcription Polymerase Chain Reaction (RT-PCR)

The Caco-2 cells were seeded in a 6-well plate (7 × 10^5^ cells per well) and stabilized for 24 h. RNA was extracted using RiboEX (GeneAll Biotechnology, Seoul, Korea) 24 h after stimulation with GB83. After quantification of the RNA concentration (1000 ng), cDNA was synthesized using RT and GO master mix (MPbio, Santa Ana, CA, USA) and oligo (dT) primers (ELPIS-Biotech, Daejeon, Korea). The template DNA (0.5–10% of the first RT reaction volume) and primers (10 pmol/µL) were added to Maxime PCR PreMix (iNtRON Biotechnology, Gyeonggi-do, Korea) tubes, and distilled water was added to prepare a total volume of 20 µL. The primer sequences were as follows: human ZO-1 (forward: 5′-TGCCATTACACGGTCCTCTG-3′, reverse: 5′-GGTTCTGCCTCATCATTTCCTC-3′); human occludin (forward: 5′-AGTGTGATAATAGTGAGTGCTATCC-3′, reverse: 5′-TGTCATACCTGTCCATCTTTCTTC-3′); human claudin-1 (forward: 5′-TTCTCGCCTTCCTGGGATG-3′, reverse: 5′-CTTGAACGATTCTATTGCCATACC-3′); human claudin-2 (forward: 5′-CAGCATTGTGACAGCAGTTG-3′, reverse: 5′-TTGGTAGGCATCGTAGTAGTTG-3′), and GAPDH (forward: 5′-CGCTCTCTGCTCCTCCTGTT-3′, reverse: 5′-CCATGGTGTCTGAGCGATGT-3′). PCR was conducted according to the manufacturer’s instructions.

### 2.4. Western Blot Analysis

The treated cells were washed with cold phosphate-buffered saline (PBS; ELPIS Biotech, Lexington, MA, USA), and proteins were harvested using a protein extraction solution (ELPIS Biotech) supplemented with protease inhibitors (Sigma-Aldrich) and phosphatase inhibitors (Roche Diagnostic, Mannheim, Germany). Protein concentration was measured using a bicinchoninic acid assay kit (Thermo Fisher Scientific, Waltham, MA, USA). The protein (10 μg) was boiled at 95 °C for 5 min after adding 5× sample buffer (ELPIS Biotech). Total protein was separated by 10% sodium dodecyl sulfate-polyacrylamide gel electrophoresis (SDS-PAGE) and transferred to polyvinylidene fluoride membranes (Merck Millipore Corporation, Billerica, MA, USA) using a wet/tank blotting system (Bio-Rad Laboratories, Hercules, CA, USA). The membranes were blocked with 5% bovine serum albumin (MP Biomedicals, Solon, OH, USA) or 5% skimmed milk (BD Biosciences, San Jose, CA, USA) in Tris-buffered saline with 0.05% Tween-20 (TBS-T, Amresco, Solon, OH, USA) for 1 h at room temperature. The membranes were then incubated overnight at 4 °C with the primary antibodies: Atg9A (1:1000), Atg16L1 (1:1000), LC3B (1:1000), Beclin-1 (1:1000) (Novus Biologicals, LLC, Centennial, CO, USA), Claudin-1 (1:1000), ZO-1 (1:1000) (Invitrogen Life Technologies), p-ERK1/2 (1:2000), ERK1/2 (1:2000) (Cell signaling Technology, Danvers, MA, USA), PAR2 (1:1000) (Proteintech Group, Inc., Rosemont, IL, USA), and β-actin (1:10,000) (Sigma-Aldrich). After incubation, the membranes were washed five times with TBS-T and then incubated with secondary antibodies at room temperature for 1–2 h. The proteins were detected using an enhanced chemiluminescence detection system (WesternBright^TM^ ECL, Advansta, Menol Park, CA, USA). Representative bands and other repeats are shown in Supplementary Figures S1–S3.

### 2.5. Immunoprecipitation (IP) Assay

Proteins were harvested in the same way as in Western blotting, and protein lysate (500 μg) was incubated overnight at 4 °C with primary antibody β-arrestin (Santa Cruz Biotechnology, Inc., Dallas, TX, USA). G PLUS-Agarose (Santa Cruz Biotechnology) was added and incubated at 4 °C on a rocker platform for 4 h. Samples were collected by centrifugation at 1000× *g* at 4 °C for 5 min, after which the supernatant was carefully removed. The pellets were washed four times with 1 mL of PBS supplemented with protease and phosphatase inhibitors. After the final wash, the supernatant was aspirated, and a 5× sample buffer was added to the pellet and boiled at 95 °C for 5 min. Protein samples were analyzed by SDS-PAGE and immunoblotting (IB).

### 2.6. Transepithelial Electrical Resistance (TEER) Measurement

The Caco-2 cells were seeded at 1 × 10^5^ cells per insert in transwell inserts with a pore size of 0.4 μm (SPL Inc., Houston, TX, USA). The cell medium was filled with 1 mL and 0.2 mL in basolateral (well) and apical (insert) compartments, respectively, and replaced with fresh medium every 2–3 days. The cells were incubated for 7 days at 37 °C in a 5% CO_2_ atmosphere. TEER was measured using a Millicell^®^ ERS-2 m (EMD Millipore, Billerica, MA, USA) such that the chopstick electrode started floating without any contact with the cells in the insert chamber. Cell-free inserts were measured as blanks, and all sample resistances were calculated by subtracting the average of the blank resistances. The unit area resistance was calculated by multiplying the resistance (Ohms) by the effective membrane area (0.3 cm^2^).

## 3. Results

### 3.1. PAR2 Inhibition Downregulated Autophagy and Reduced Intestinal Barrier Function in Caco-2 Cells

**Figure 1:**
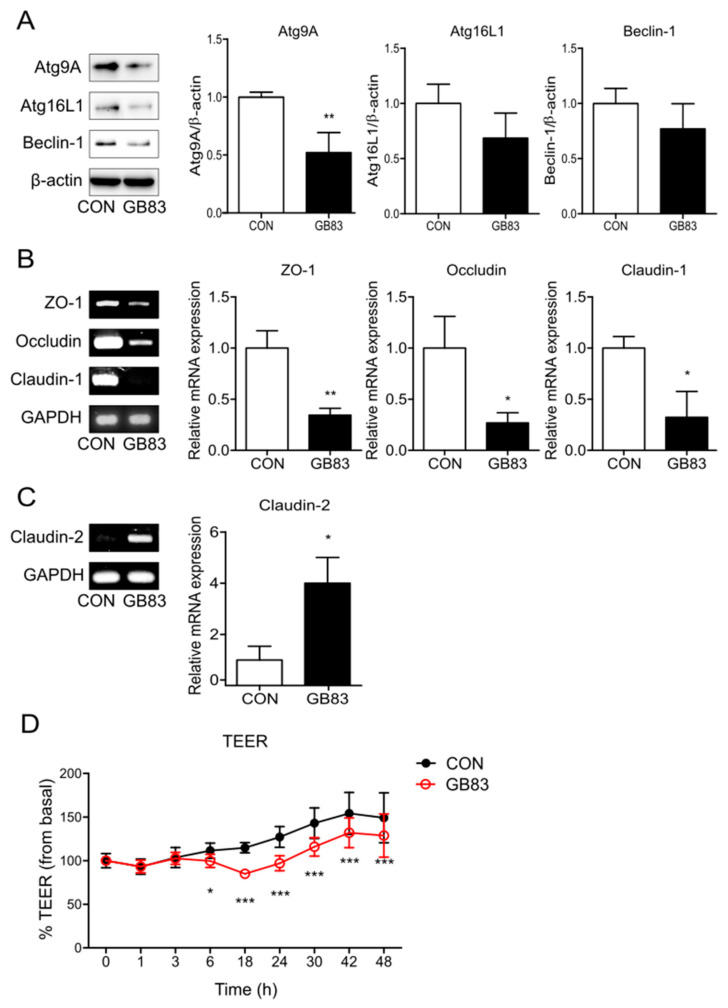
Inhibition of PAR2 decreased the expression of autophagy- and TJ-related factors and increased permeability. Cells were pretreated with SFM for 24 h and treated with GB83 (10 µM) for 24 h. (**A**) The protein levels of autophagy-related proteins were detected using Western blotting. β-actin was used as a loading control. The column bar graph represents the ratio of each protein to β-actin. mRNA expressions of (**B**) ZO-1, occludin, claudin-1, and (**C**) claudin-2 in Caco-2 cells treated with GB83 were detected using RT-PCR. GAPDH was used as the loading control. The bar graph represents the ratio of each mRNA to GAPDH. Data are shown as mean ± SD. * *p* < 0.05 and ** *p* < 0.01 by unpaired t-test compared to the control group. (**D**) Caco-2 cells were cultured on transwell inserts for 7 days, pretreated with SFM for 24 h, and then treated with GB83 (10 µM). TEER was measured at different time points for 48 h after GB83 treatment. Values are mean ± SD. * *p* < 0.05, *** *p* < 0.001 by two-way ANOVA compared to the control group.

To investigate whether inhibition of PAR2 downregulates autophagy in the intestinal epithelial cells, changes in the expression of autophagy-related proteins were evaluated using Western blotting. The expressions of autophagy markers (Atg9, Atg16L1, and beclin-1) were lower in Caco-2 cells when PAR2 was inhibited using GB83, a PAR2 antagonist (Figure 1A). These results suggest that PAR2 inhibition blocks autophagy.

Activation of PAR2 on epithelial cells may affect tight-junction permeability [28]. ZO-1 plays a role in linking the actin cytoskeleton and transmembrane proteins, and claudin-1 and occludin are transmembrane proteins with a barrier function that form TJs. Claudin-2 is permeable to both water and ions and increased expression of claudin-2 is associated with increased permeability [11]. To observe the change in TJs caused by PAR2, the expression of TJ proteins was evaluated by inhibiting PAR2. This inhibition decreased the mRNA levels of TJs (ZO-1, occludin, and claudin-1; Figure 1B) and increased the mRNA level of claudin-2 (Figure 1C). These results suggest that PAR2 strengthens the TJs in the intestinal epithelial cells. In addition, as the alterations in TJ protein expression are highly related to intestinal epithelial permeability, TEER measurement is required to determine whether PAR2 regulation affects intestinal permeability. PAR2 inhibition reduced intestinal epithelial resistance (Figure 1D). These results indicate that PAR2 enhances TJs to decrease mucosal permeability.

### 3.2. PAR2 Prolonged ERK1/2 Phosphorylation through the Assembly of the PAR2-ERK1/2-β-Arrestin Complex

**Figure 2:**
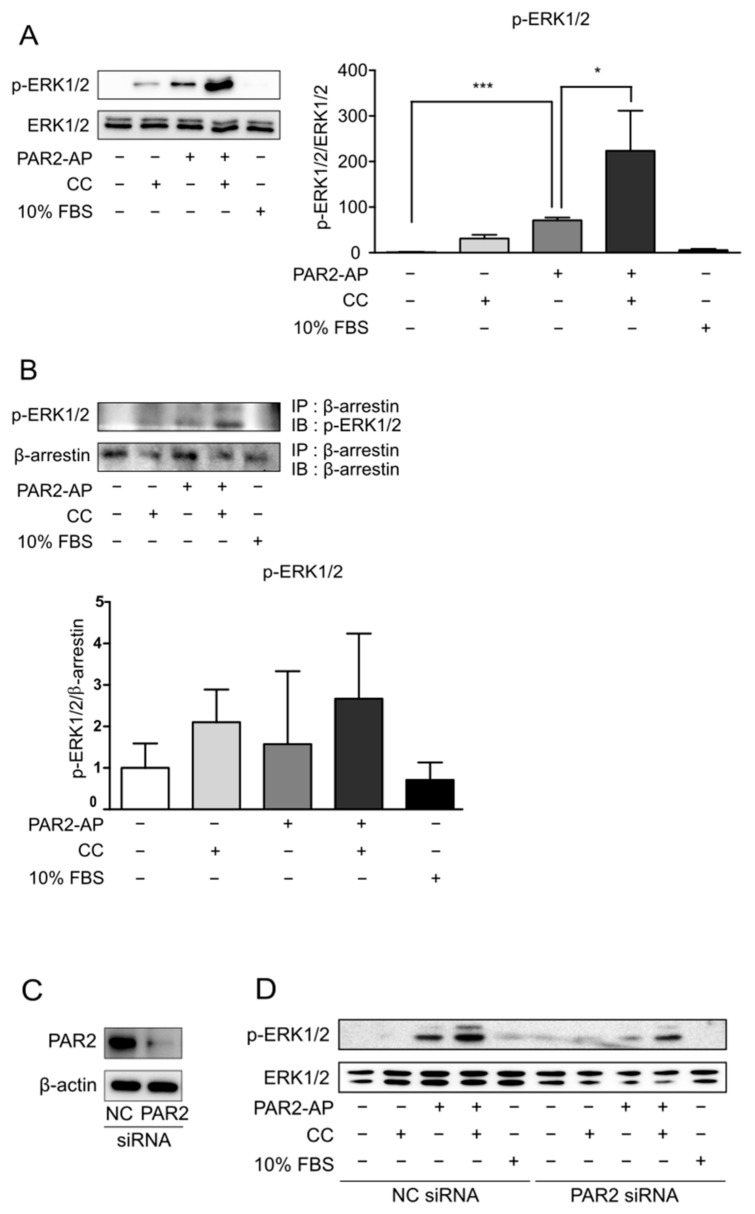
PAR2 activation led to the recruitment of β-arrestin and ERK1/2. Cells were serum-starved for 24 h and treated with PAR2-AP (100 µM) or CC (IL-1β, 1 ng/mL; TNF-α, 20 ng/mL; and IFN-γ, 10 ng/mL) or 10% FBS for 6 h. (**A**) The protein level of p-ERK1/2 was measured using Western blotting. The bar graph indicates the ratio of p-ERK1/2 to ERK1/2. Data are shown as mean ± SD. * *p* < 0.05, *** *p* < 0.001 (one-way ANOVA). (**B**) Cell lysates were immunoprecipitated with β-arrestin antibody, followed by IB with p-ERK1/2 antibody. The bar graph indicates the ratio of p-ERK1/2 to β-arrestin. Data are shown as mean ± SD. Caco-2 cells were transfected with PAR2 siRNA for 48 h and then treated with PAR2-AP (100 µM) or CC (IL-1β, 1 ng/mL; TNF-α, 20 ng/mL; and IFN-γ, 10 ng/mL) or 10% FBS for 6 h. (**C**) Western blotting was used to detect a decrease in PAR2 protein expression in PAR2-KD cells. (**D**) p-ERK1/2 protein levels in PAR2-KD cells were measured using Western blotting.

There are various pathways through which PAR2 activates ERK1/2 [2]. Additionally, it has been demonstrated that ERK1/2 recruitment through β-arrestin prolongs ERK1/2 activity [23]. Therefore, we hypothesized that PAR2 prolongs ERK1/2 activity through β-arrestin signaling in an inflammatory environment. To test this hypothesis, the protein level of p-ERK1/2 was measured using Western blot analysis. Caco-2 cells were pre-treated with SFM for 24 h to eliminate the background caused by the serum. CC indicates an inflammatory condition, and 10% FBS represents a condition in which ERK1/2 is generally activated. ERK1/2 activity did not persist in the control or 10% FBS groups. However, the activation of ERK1/2 was prolonged by PAR2-AP and was prolonged when both CC and PAR2-AP were used (Figure 2A). This result indicates that the activation of PAR2 in an inflammatory environment prolongs ERK1/2 activation. Moreover, an interaction between p-ERK1/2 and β-arrestin was observed in the CC and PAR2-AP treatments (Figure 2B). In other words, PAR2 activation in the inflammatory environment leads to the assembly by recruitment of β-arrestin and ERK1/2.

Next, to test whether PAR2 recruits ERK1/2 and prolongs its activation, we performed PAR2-KD and observed the changes in p-ERK1/2. PAR2 siRNA was transfected into the Caco-2 cells. Negative control cells and PAR2-KD cells were each treated with PAR2-AP for PAR2 activation and CC treatment for the inflammatory environment, and 10% FBS treatment was performed to check whether ERK1/2 was normally activated. PAR2-KD was verified using Western blotting (Figure 2C). The protein expression level of p-ERK1/2 in PAR2-KD cells was lower than that in the control (Figure 2D), indicating prolonged activation of ERK1/2 by PAR2.

### 3.3. Enhanced Autophagy Signaling by PAR2 Was Related to ERK1/2 Phosphorylation

**Figure 3:**
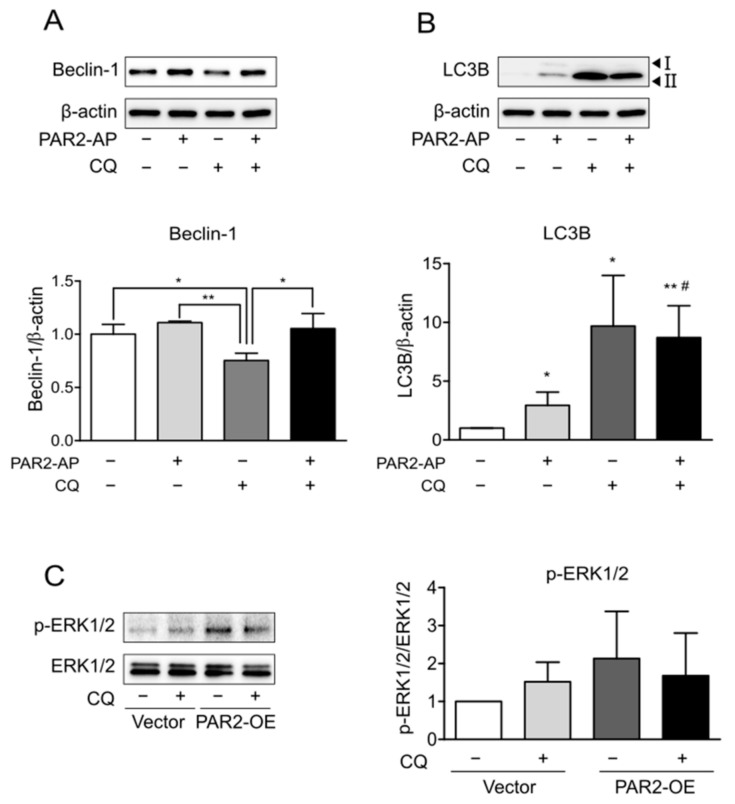
The protein level of p-ERK1/2 was reduced by autophagy inhibition. Caco-2 cells were serum-starved for 24 h and treated with PAR2-AP (100 µM) or CQ (100 µM) for 24 h. β-actin was used as the loading control. The column bar graph signifies the ratio of each protein to β-actin. Data are shown as mean ± SD. (**A**) Western blotting was used to measure the protein level of beclin-1. * *p* < 0.05, ** *p* < 0.01 with one-way ANOVA. (**B**) The expression of LC3B was detected using Western blotting. * *p* < 0.05, ** *p* < 0.01 compared to the control group; # *p* < 0.05 compared to the PAR2-AP group (one-way ANOVA). Caco-2 cells were transfected with vector plasmid and PAR2-OE plasmid for 48 h and then treated with CQ (100 µM). (**C**) Western blotting was used to detect p-ERK1/2 protein in PAR2-OE cells. The bar graph indicates the ratio of p-ERK1/2 to ERK1/2. Data are shown as mean ± SD.

CQ blocks autophagic flux by inhibiting the autophagosome-lysosome fusion step in autophagy [29]. First, the expression of autophagy markers was observed by the inhibition of autophagy by CQ. The expression of beclin-1 decreased due to CQ (Figure 3A, lane 3), and that of accumulated LC3B increased (Figure 3B, lane 3). In addition, PAR2-AP and CQ cotreatment reduced beclin-1 expression to a lesser extent than CQ treatment alone (Figure 3A, lane 4). These results indicated that activation of PAR2 ameliorated CQ-induced inhibition of autophagy.

PAR2 prolonged ERK1/2 phosphorylation via the β-arrestin mechanism (Figure 2). To determine whether this signaling is related to PAR2-induced autophagy, ERK1/2 activity was observed after the inhibition of autophagy. Prolonged ERK1/2 phosphorylation by PAR2 activation decreased upon suppression of autophagy (Figure 3C). This result suggests that PAR2 prolongs ERK1/2 activity, and this is associated with enhanced autophagy signaling by PAR2.

### 3.4. PAR2 Regulated Epithelial Permeability by Autophagy

**Figure 4:**
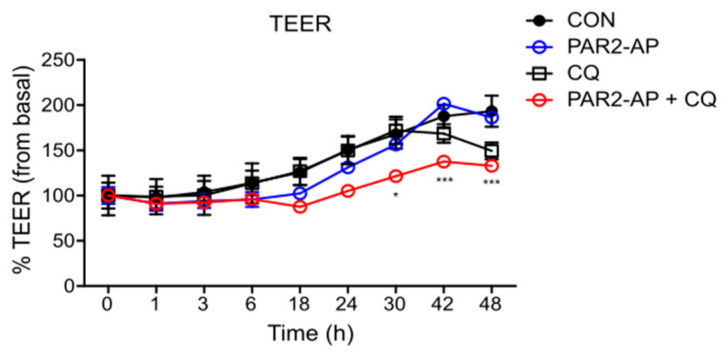
PAR2 modulated TJ and increased intestinal barrier permeability when autophagy was inhibited. Caco-2 cells were cultured on transwell inserts for 7 days, pretreated with SFM for 24 h, and then treated with PAR2-AP (100 µM) or CQ (10 µM). TEER was measured at different time points for 48 h after treatment. Values are mean ± SD. * *p* < 0.05, *** *p* < 0.001 compared to the PAR2-AP group (two-way ANOVA).

To examine whether autophagy is required for PAR2 to regulate intestinal mucosal permeability, TEER was measured by inhibiting autophagy in Caco-2 cells. A decrease in TEER value was observed after treatment with PAR2-AP and CQ (Figure 4), indicating that an activated PAR2 maintains mucosal integrity through autophagy.

## 4. Discussion

Although PAR2 is distributed throughout the GI tract and is involved in various GI functions, it plays a dual role in intestinal inflammation [30]. PAR2 reduces metabolic dysfunction by inducing autophagy in a high-fat environment and improves intestinal inflammation [24]. However, the association between PAR2-regulated autophagy and TJ regulation has not yet been elucidated. In this study, we found that PAR2 regulates intestinal epithelial TJs by inducing autophagy, which contributes to the maintenance of intestinal homeostasis by regulating mucosal barrier permeability.

Consistent with a previous study [24], PAR2 was found to induce autophagy. Inhibition of PAR2 decreased the expression of proteins involved in autophagosome formation and maturation during autophagy (Figure 1A). In addition, PAR2 strengthened the intestinal epithelial barrier function. Inhibition of PAR2 reduced the expression of factors such as ZO-1, occludin, and claudin-1, which constitute the selective barrier (Figure 1B). On the contrary, claudin-2 was upregulated in the small and large intestines and induced diarrhea through a leaky flux mechanism [31]. Inhibition of PAR2 increased the expression of claudin-2 (Figure 1C), i.e., PAR2 increased the expression of factors that enhance barrier function and decreased the expression of factors that induce functional decline. Among others, PAR2 increased TJ formation and thus decreased intestinal mucosal permeability. When PAR2 was inhibited, the epithelial resistance decreased in enterocytes (Figure 1D). These results are contrary to previous findings that PAR2 activation reduces intestinal epithelial resistance [25]. This opposing effect may be associated with PAR2-mediated autophagy, which maintains intestinal homeostasis. Our previous study showed that PAR2 activation induced autophagy, and this reduced intestinal inflammation accompanied by a high-fat environment [24]. In addition, autophagy strengthens the mucosal barrier function in the intestine [27]. Therefore, PAR2 inhibition may weaken the barrier function and disturb intestinal homeostasis. These reports and our results suggest that PAR2-mediated autophagy can contribute to maintaining mucosal barrier integrity and intestinal stability.

PAR2 signaling occurs through the G-protein or β-arrestin pathways. β-arrestin induces PAR2 internalization to form a complex, induces ERK1/2 activation, and delays its translocation to the nucleus [23]. This signaling was found to be more induced in an inflammatory environment (Figure 2A,B). When forming the PAR2–β-arrestin–ERK1/2 complex, ERK1/2 was recruited after PAR2 activation (Figure 2D). As activation of PAR2 has already been shown to regulate TJ through the β-arrestin–ERK1/2-dependent pathway [23], we assumed that TJ regulation of autophagy by PAR2 also occurred through β-arrestin signaling. When autophagy was inhibited, PAR2 activation increased the signal of the repressed autophagy. The expression of beclin-1, an autophagy-related protein that was reduced by autophagy inhibition, increased after PAR2 activation (Figure 3A). An autophagy inhibitor (CQ) inhibits autophagosome formation during the autophagy process [29]. LC3B is a structural component of the autophagosome and, therefore, is involved in autophagosome membrane expansion and fusion to form LC3B-containing autophagosome complex [32]. CQ inhibits autophagosome formation during the autophagy process [29]. Since CQ blocks autophagosome–lysosome fusion, CQ treatment induces accumulation of LC3B during autophagy induction [32]. Therefore, measuring the accumulation of LC3B can monitor autophagy inhibition in different cell types [32,33,34]. Thus, it appears that autophagosome degradation is inhibited and accumulated and thereby increases the expression of LC3B (Figure 3B). Moreover, inhibition of autophagy with CQ decreased the activity of p-ERK1/2, which was prolonged by PAR2 (Figure 3C). These results suggest that enhanced autophagy signaling by PAR2 is related to the β-arrestin–ERK1/2 pathway.

We determined whether autophagy is involved in the regulation of TJs by PAR2. We observed that the autophagy signal induced by the activation of PAR2 regulated intestinal epithelial permeability. Inhibition of autophagy in the Caco-2 cells did not affect intestinal epithelial resistance. The intestinal epithelial resistance value maintained during the activation of PAR2 was significantly reduced when autophagy was suppressed (Figure 4). Autophagy is necessary to maintain antibacterial defenses and mucosal immune responses [6]. The intestinal epithelium is a single-cell layer that constitutes the most important barrier and acts as a selective permeability barrier to maintain defense [17]. In other words, PAR2 induces autophagy, suggesting that this signal helps maintain an optimal intestinal environment by regulating intestinal epithelial resistance.

**Figure 5:**
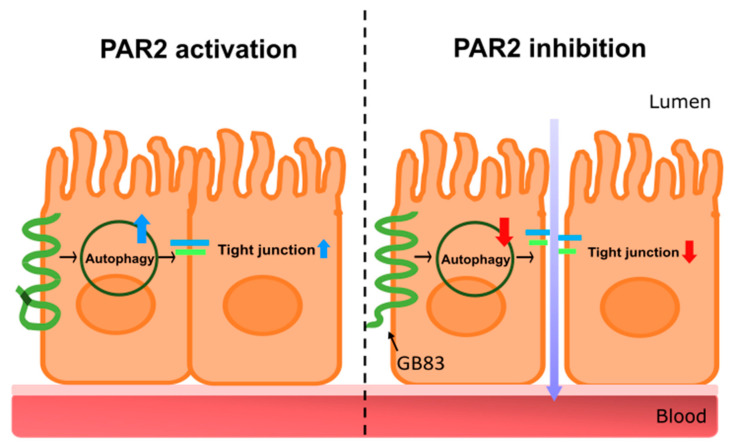
A graphical representation of intestinal barrier regulation through autophagy regulation by PAR2. The graphic is a summary of the research results. The left shows PAR2-activated signaling. Activation of PAR2 induces autophagy, upregulating tight junctions, and maintaining intestinal integrity. PAR2 is involved in the regulation of the homeostasis of the intestinal epithelial mucosa. The right shows signaling when PAR2 is inhibited. Inhibition of PAR2 reduces autophagy, which downregulates tight junctions and loosens the connections between intestinal epithelial cells. This results in a decrease in intestinal epithelial resistance, an increase in intestinal permeability, and the influx of external agents such as toxins, food debris, and pathogens. PAR2, protease-activated receptor 2.

In conclusion, this study demonstrated that PAR2 in the intestinal epithelial cells is essential for maintaining intestinal mucosal integrity by enhancing autophagy (Figure 5). These findings may further contribute to the development of PAR2-targeted therapeutics that enhance autophagy to improve leaky gut and intestinal inflammation.

## Data Availability

Data sharing not applicable.

## Supplementary Materials

The following supporting information can be downloaded at: https://www.mdpi.com/article/10.3390/cells11050878/s1, Figure S1: Western blotting in Figure S1A was repeated 3 times and RT-PCR in Figure S1B,C were repeated 3 times, Figure S2: Western blotting in Figure S2A was repeated 3 times and IP in Figure S2B was repeated 3 times, Figure S3: Western blotting in Figure S3A–D were repeated 3 times.
