# Supplementary figures and images for: Modulation of Intestinal Epithelial Permeability via Protease-Activated Receptor-2-Induced Autophagy
Source: Cells. 2022 Mar 3;11(5):878. doi: 10.3390/cells11050878 (PMC8909592; doi:10.3390/cells11050878)

**A**

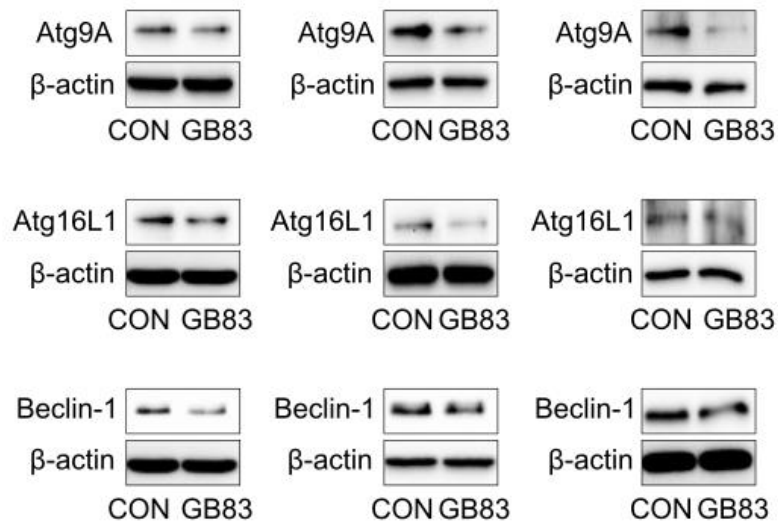

**B**

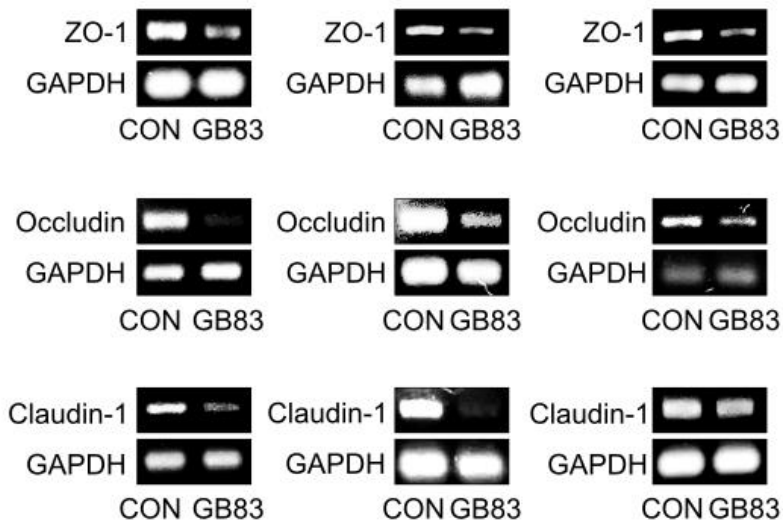

**C**

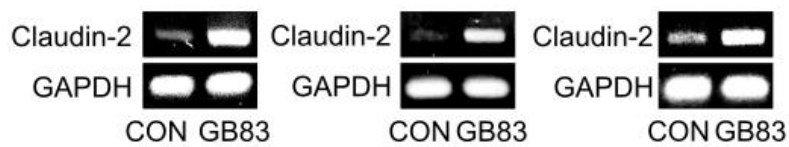

Supplementary Figure S1

**A**

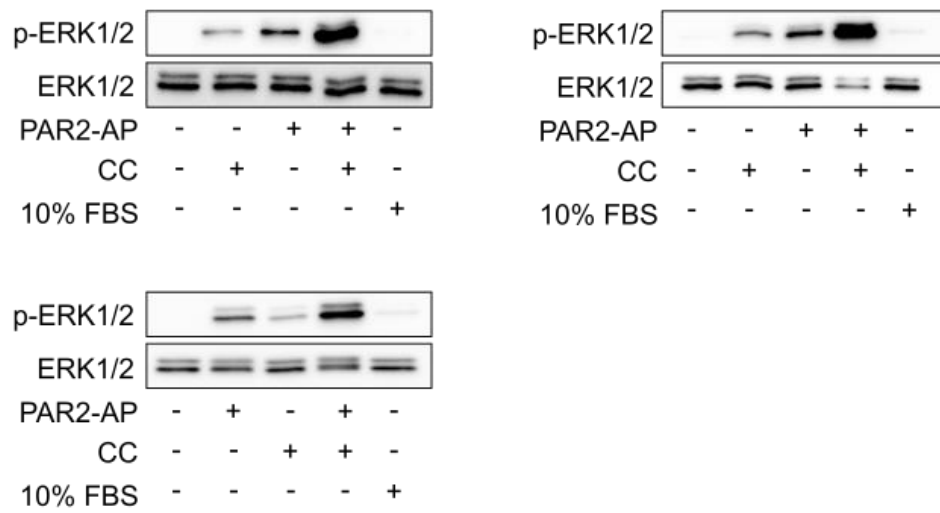

**B**

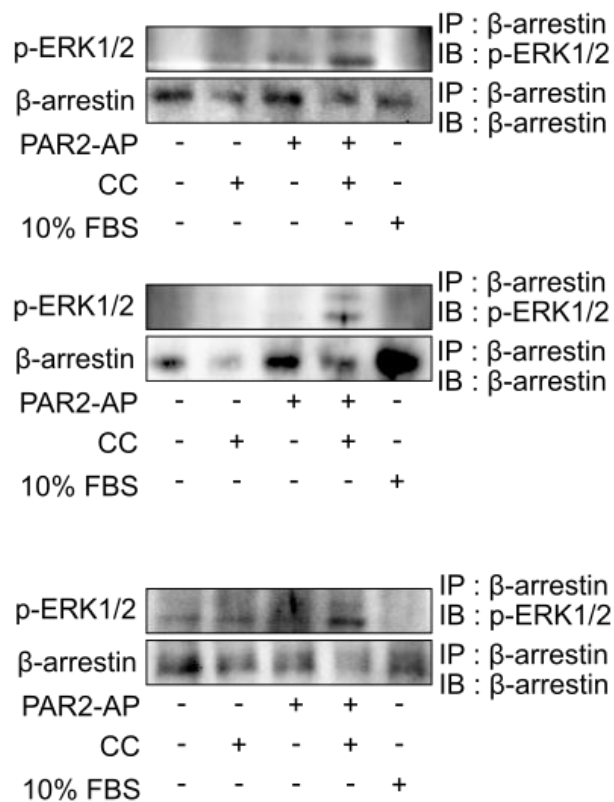

Supplementary Figure S2

A

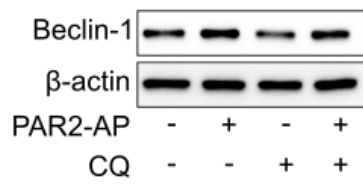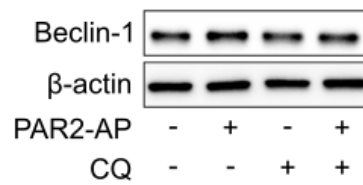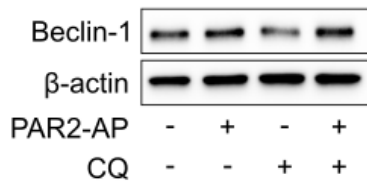

B

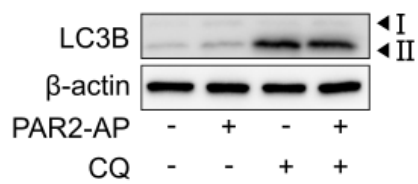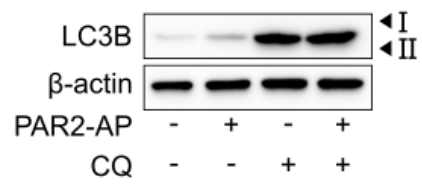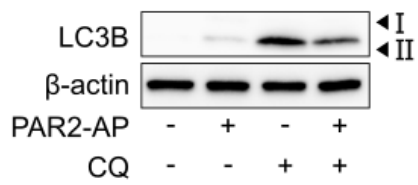

C

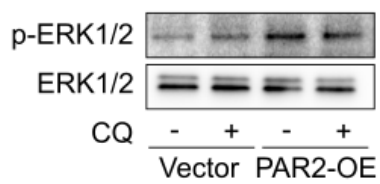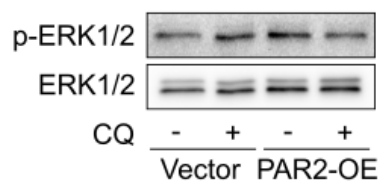

D

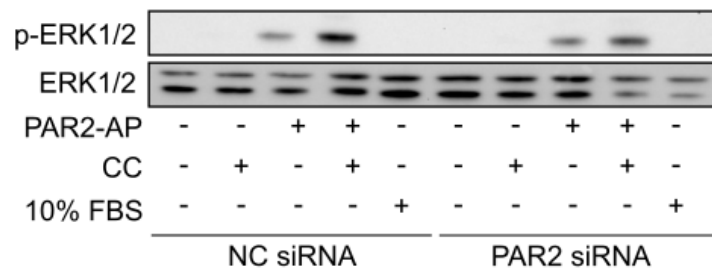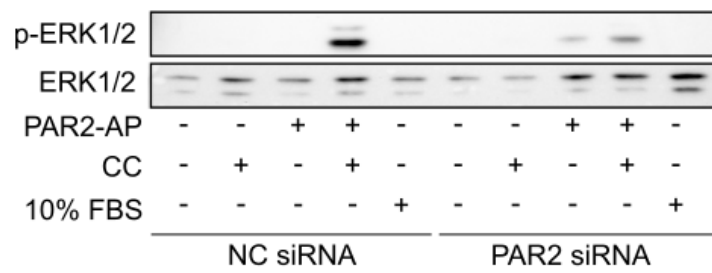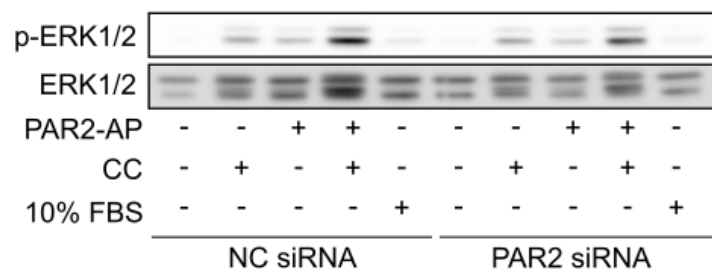

Supplementary Figure S3

Supplement: Supplementary file 1 [file cells-11-00878-s001.zip › cells-1545964-supplementary.pdf]
